# Draft Genome Sequences of 59 Endospore-Forming Gram-Positive Bacteria Associated with Crop Plants Grown in Vietnam

**DOI:** 10.1128/MRA.01154-20

**Published:** 2020-11-19

**Authors:** Le Thi Thanh Tam, Jennifer Jähne, Pham Thi Luong, Le Thi Phuong Thao, Le Thi Kim Chung, Andy Schneider, Christian Blumenscheit, Peter Lasch, Thomas Schweder, Rainer Borriss

**Affiliations:** a Division of Plant Pathology and Phyto-Immunology, Plant Protection Research Institute, Hanoi, Vietnam; b Proteomics and Spectroscopy Unit (ZBS6), Centre for Biological Threats and Special Pathogens, Robert Koch Institute, Berlin, Germany; c Labo Center, Institute for Preventive Medicine and Public Health, Hanoi Medical University, Hanoi, Vietnam; d Institute of Marine Biotechnology e.V., Greifswald, Germany; e Nordreet UG, Greifswald, Germany; University of Maryland School of Medicine

## Abstract

We report the draft genome sequences of 59 Gram-positive bacterial strains that were isolated from Vietnamese crop plants. The strains were assigned to nine different *Bacillus* and *Brevibacillus* species. Ten strains classified as being a *Bacillus* sp. (3 strains), *Brevibacillus* sp. (6 strains), or *Lysinibacillus* sp. (1 strain) could not be identified to the species level.

## Abstract (precis)

We report the draft genome sequences of 59 Gram-positive bacterial strains that were isolated from Vietnamese crop plants. The strains were assigned to nine different *Bacillus* or *Brevibacillus* species. Ten strains classified as being *Bacillus* spp. (3 strains), *Brevibacillus* spp. (6 strains), or *Lysinibacillus* sp. (1 strain) could not be identified to the species level.

## ANNOUNCEMENT

Endophytic and plant-associated Gram-positive bacteria were isolated from Vietnamese crop plants such as coffee, black pepper, maize, orange trees, dragon trees, tomato, and cabbage (for details, see Table 1). Samples were obtained from the soil adjacent to plant roots, the surface, and the inner tissue of different plant parts such as root, stem, and leaf (Table 1). Samples from inner tissues were propagated after surface sterilization using 70% ethanol and 1% sodium hypochlorite (1). *Brevibacillus* spp. were found to be enriched when soil samples adherent to plant roots were incubated with shaking for a further 2 weeks. Diluted samples were incubated on either half-strength tryptic soy broth or tryptone-yeast extract-glucose agar (2) for 3 to 5 days at 30°C. In order to enrich endospore-forming bacteria, single colonies were picked from agar plates, diluted in 0.5 ml 0.9% NaCl, and heat treated for 20 min at 80°C. Only strains that were able to suppress fungal plant pathogens, such as Fusarium oxysporum, *Phytophthora palmivora*, or *Neoscytalidium dimidiatum*, under *in vitro* conditions were used in further experiments. As a first step for characterizing these strains more completely, the isolates underwent genome sequencing, and their taxonomy based on their draft genome sequences was evaluated.

**TABLE 1:** List of the 59 endospore-forming Gram-positive bacteria isolated from Vietnamese crop plants

GenBank accession no.	SRA accession no.	Sample name	Estimated genome size (bp)	G+C content (%)	Total no. of raw reads	Genome coverage (×)	No. of contigs	Contig *N*_50_ (bp)	Total no. of genes	Isolation source and date (day-mo-yr)	Taxonomy according to dDDH and ANIb results	dDDH (%)^*a*^	ANIb (%)^*b*^	Similar type strain
JABMIW000000000	SRR12133238	BT2.2	3,708,421	44.0	7,888,450	28.0	22	556,510	3,798	Dragon tree stem, 5-5-2019	B. altitudinis	86.20	98.26	*B. altitudinis* DSM 21631
VDDR00000000	SRR12104977	A8	5,071,716	35.4	5,427,083	52.0	52	307,045	5,877	Coffee root, 1-9-2018	B. cereus	90.50	98.74	B. cereus ATCC 14579
VEPO00000000	SRR12104976	A22	5,812,849	35.2	5,059,544	56.0	57	315,527	6,032	Coffee root, 1-9-2018	B. cereus	74.20	96.80	B. cereus ATCC 14579
VEPP00000000	SRR12104975	A24	5,689,021	35.7	2,398,472	28.0	73	233,470	5,897	Black pepper root, 1-9-2018	B. cereus	85.50	98.13	B. cereus ATCC 14579
VEPT00000000	SRR12283339	A31	5,319,678	35.3	5,328,004	40.0	102	246,002	5,556	Coffee rhizosphere, 1-9-2018	B. cereus	90.10	98.64	B. cereus ATCC 14579
VEPQ00000000	SRR12104956	A42	5,604,011	35.6	6,770,872	80.0	42	489,606	5,708	Black pepper root, 1-9-2018	B. cereus	74.30	96.75	B. cereus ATCC 14579
JABSVB000000000	SRR12115356	HB3.1	5,796,358	34.8	5,938,348	15.0	82	478,132	5,867	Orange plant adherent soil, 17-4-2019	B. cereus	89.80	98.25	B. cereus ATCC 14579
JABSVE000000000	SRR12132980	HD1.4B	5,660,925	34.9	5,561,352	13.0	53	831,879	5,737	Tomato root soil, 23-4-2019	B. cereus	88.40	98.20	B. cereus ATCC 14579
JABSVC000000000	SRR12124346	HD2.4	5,660,820	34.9	8,250,328	20.0	48	378,831	5,734	Tomato root adherent soil, 23-4-2019	B. cereus	88.30	98.22	B. cereus ATCC 14579
JABSVF000000000	SRR12141689	M2.1B	5,869,336	34.8	8,131,108	20,0	63	344,459	6,029	Maize field soil, 6-12-2018	B. cereus	73.10	96.28	B. cereus ATCC 14579
VEPR00000000	SRR12104981	SN4.3	5,594,617	35.6	3,571,304	45.0	78	146,072	5,773	*Ostrinia nubilalis*, 1-11-2018	B. cereus	73.60	96.76	B. cereus ATCC 14579
VEPS00000000	SRR12042680	TK1	6,195,299	34.7	11,965,210	30.0	363	83,852	6,483	Coffee rhizosphere, 1-11-2018	B. cereus	73.50	95.96	B. cereus ATCC 14579
VEPU00000000	SRR12104980	SN4.1	5,443,801	35.2	4,526,204	40.0	32	868,705	5,590	*Ostrinia nubilalis*, 1-11-2018	*B. pacificus*	77.20	97.10	*B. pacificus* EB422
VEPY00000000	SRR12104982	CD3-1a	5,150,560	35.2	4,168,978	56.0	40	589,852	5,431	*Brassica juncea* rhizosphere, 1-11-2018	*Bacillus* sp.	62.50	94.31	*B*. *tropicus* N24
VEPX00000000	SRR12104984	CD3-5	5,695,940	35.3	4,593,634	60.0	50	302,092	5,784	*Brassica juncea* rhizosphere, 1-11-2018	*Bacillus* sp.	69.60	95.45	*B. pacificus* EB422
JABSVD000000000	SRR12132949	HD1.3	5,695,940	35.1	7,791,502	19.0	56	469,972	5,784	Tomato root soil, 23-4-2019	*Bacillus* sp.	66.30	95.04	*B. pacificus* EB422
JABMIX000000000	SRR12141623	GR2.1	4,084,062	43.6	3,512,065	25.0	22	1,042,180	4,240	Green egg-plant root, 6-12-2018	B. subtilis	88.50	98.49	B. subtilis ATCC 6051
JABMIY000000000	SRR12142185	DL2.1	3,927,651	44.0	3,260,262	24.0	46	476,949	4,159	Maize field soil, 8-5-2018	*B. tequilensis*	78.50	97.25	*B. tequilensis* KCTC 13622
VEPW00000000	SRR12104983	CD3-2	5,958,606	35.8	2,788,296	30.0	68	236,159	6,186	*Brassica juncea* rhizosphere, 1-11-2018	*B. tropicus*	72.40	96.68	*B*. *tropicus* N24
VEPV00000000	SRR12104979	SN1	5,335,513	35.2	5,015,568	63.0	34	456,889	5,560	*Ostrinia nubilalis*, 1-11-2018	*B. tropicus*	72.50	96.70	*B*. *tropicus* N24
VEWT00000000	SRR12132949	A25	3,833,140	46.2	5,120,982	88.0	25	981,821	3,834	Black pepper root, 1-9-2018	*B. velezensis*	79.90	96.41	*B. velezensis* KCTC 13012
VEWU00000000	SRR12104957	A35	3,867,857	46.3	5,469,886	93.0	20	561,718	3,896	Black pepper root, 1-9-2018	*B. velezensis*	79.60	96.48	*B. velezensis* KCTC 13012
JABSVZ000000000	SRR12142193	BP1.2A	3,872,427	46.4	7,443,818	30.0	30	471,958	3,832	Sand in Mooc River, 8-5-2019	*B. velezensis*	96.20	98.16	*B. velezensis* KCTC 13012
JABSVT000000000	SRR12133237	BT2.1	3,889,067	46.4	13,578,068	50.0	33	475,102	3,855	Dragon tree stem, 8-5-2019	*B. velezensis*	85.80	98.23	*B. velezensis* KCTC 13012
JABSVU000000000	SRR12133171	BT2.4	3,865,088	46.4	3,355,802	12.0	46	422,236	3,851	Dragon tree stem, 8-5-2019	*B. velezensis*	95.30	98.24	*B. velezensis* KCTC 13012
JABSVG000000000	SRR12113939	CP5.2	3,932,265	46.4	4,591,652	17.0	34	563,859	3,876	Orange plant root, 17-4-2019	*B. velezensis*	80.30	97.25	*B. velezensis* KCTC 13012
JABSVH000000000	SRR12114569	CP6	3,825,619	46.7	6,766,126	19.0	34	482,295	3,782	Orange plant leaf, 17-4-2019	*B. velezensis*	81.10	97.46	*B. velezensis* KCTC 13012
JABSVI000000000	SRR12115431	CP7.1A	3,874,748	46.4	4,658,644	17.0	36	475,102	3,836	Orange plant soil, 17-4-2019	*B. velezensis*	96.40	98.04	*B. velezensis* KCTC 13012
JABSVJ000000000	SRR12123548	CP7.1C	3,871,413	46.4	4,273,868	16.0	45	339,955	3,840	Orange plant soil, 17-4-2019	*B. velezensis*	96.20	98.04	*B. velezensis* KCTC 13012
JABSVK000000000	SRR12123552	CP7.2A	3,883,818	46.4	4,191,800	16.0	34	468,775	3,857	Orange plant soil, 17-4-2019	*B. velezensis*	85.80	98.03	*B. velezensis* KCTC 13012
JABSVM000000000	SRR12123706	DP1.3B	3,877,050	46.4	4,778,546	18.0	24	486,178	3,827	Orange plant soil, 17-4-2019	*B. velezensis*	96.70	98.03	*B. velezensis* KCTC 13012
JABSVL000000000	SRR12123599	DP2.2B	3,871,310	46.4	4,996,856	18.0	40	471,958	3,840	Orange plant soil, 17-4-2019	*B. velezensis*	96.20	98.04	*B. velezensis* KCTC 13012
JABSVX000000000	SRR12134139	EG5.1A	4,030,371	46.2	9,630,302	35.0	36	451,426	3,954	White egg-plant root, 6-12-2018	*B. velezensis*	92.20	98.93	*B. velezensis* KCTC 13012
JABSVP000000000	SRR12132923	HD1.1	3,875,639	46.4	4,494,558	16.0	33	475,102	3,834	Tomato root soil, 23-4-2019	*B. velezensis*	96.50	98.03	*B. velezensis* KCTC 13012
JABSVN000000000	SRR12123774	HD2.2	3,874,124	46.4	3,903,166	15.0	41	475,603	3,842	Tomato root soil, 23-4-2019	*B. velezensis*	96.40	98.04	*B. velezensis* KCTC 13012
JABSVO000000000	SRR12132925	HD2.5	3,878,653	46.4	4,716,380	17.0	37	428,165	3,861	Tomato root soil, 23-4-2019	*B. velezensis*	98.30	98.03	*B. velezensis* KCTC 13012
JABSVQ000000000	SRR12132982	HD3.1B	3,891,722	46.4	4,185,344	15.0	49	356,004	3,869	Tomato root soil, 23-4-2019	*B. velezensis*	98.20	98.04	*B. velezensis* KCTC 13012
JABSVR000000000	SRR12132999	HD5.1	3,874,949	46.4	1,960,848	14.0	48	475,102	3,852	Tomato root soil, 23-4-2019	*B. velezensis*	96.40	98.06	*B. velezensis* KCTC 13012
JABSVS000000000	SRR12133003	HD5.2A	3,878,897	46.4	6,893,794	25.0	37	428,165	3,861	Tomato leaves, 23-4-2019	*B. velezensis*	98.30	98.22	*B. velezensis* KCTC 13012
VEWV00000000	SRR12104919	KT1	3,950,573	46.4	5,413,752	90.0	40	280,310	3,970	Black pepper root, 1-9-2018	*B. velezensis*	84.70	96.13	*B. velezensis* KCTC 13012
JABSVY000000000	SRR12142183	MR2.1A	4,027,596	46.2	6,220,582	23.0	41	451,426	3,956	Maize root, 6-12-2018	*B. velezensis*	92.20	98.93	*B. velezensis* KCTC 13012
JABSVV000000000	SRR12133236	OL1.1	3,921,211	46.4	3,249,582	12.0	34	1,032,611	3,850	Orange plant leaf, 13-6-2019	*B. velezensis*	80.20	97.48	*B. velezensis* KCTC 13012
JABSVW000000000	SRR12133982	OR2.1	3,864,359	46.4	8,863,958	30.0	33	428,160	3,834	Orange plant soil, 13-6-2019	*B. velezensis*	95.30	98.25	*B. velezensis* KCTC 13012
VEWW00000000	SRR12104916	S1	3,866,305	46.1	6,223,108	106	30	486,485	3,893	Coffee rhizosphere, 1-9-2018	*B. velezensis*	85.80	97.01	*B. velezensis* KCTC 13012
VEWX00000000	SRR12104917	S2	3,864,301	46.1	6,033,960	103	33	397,838	3,893	Coffee rhizosphere, 1-9-2018	*B. velezensis*	85.70	97.12	*B. velezensis* KCTC 13012
VEWY00000000	SRR12105026	TK2	4,044,692	46.2	4,395,588	71.0	48	808,279	4,144	Field soil, 1-11-2018	*B. velezensis*	79.80	96.26	*B. velezensis* KCTC 13012
VEWZ00000000	SRR12104978	TL7	3,865,047	46.4	4,381,374	77.0	29	428,384	3,879	Coffee rhizosphere, 1-9-2018	*B. velezensis*	85.70	97.94	*B. velezensis* KCTC 13012
JABSUV000000000	SRR12113944	HB2.2	6,346,173	47.2	5,448,236	13.0	137	133,518	5,928	Orange plant soil, 17-4-2019	*Brevibacillus* sp.	53.70	93.46	Brevibacillus formosus NRRL NRS-863
JABSVA000000000	SRR12142184	RS1.1	6,246,682	47.2	4,526,908	11.0	46	810,856	5,867	Maize field soil, 8-5-2019	*Brevibacillus* sp.	54.20	93.10	*Brevibacillus formosus* NRRL NRS-863
JABSUW000000000	SRR12132961	HD1.4A	6,032,732	52.2	4,713,548	11.0	120	286,241	5,720	Tomato root soil, 23-4-2019	*Brevibacillus parabrevis*	93.80	99.07	*Brevibacillus parabrevis* 605
JABSUX000000000	SRR12132995	HD3.3A	6,074,516	52.1	4,338,330	11.0	178	190,049	5,781	Tomato root soil, 23-4-2019	*Brevibacillus parabrevis*	93.60	99.05	*Brevibacillus parabrevis* 605
JABMIV000000000	SRR12105150	HB1.1	6,317,805	47.2	6,867,506	28.0	44	376,218	6,010	Orange plant soil, 17-4-2019	*Brevibacillus porteri*	82.70	97.36	*Brevibacillus porteri* B-41110
JABMIU000000000	SRR12105325	HB1.2	6,342,770	47.1	6,815,744	17.0	55	468,289	6,077	Orange plant soil, 17-4-2019	*Brevibacillus porteri*	82.80	97.38	*Brevibacillus porteri* B-41110
JABSUT000000000	SRR12113913	HB1.4B	6,377,995	47.2	12,209,194	29.0	41	493,935	6,133	Orange plant soil, 17-4-2019	*Brevibacillus porteri*	82.20	97.24	*Brevibacillus porteri* B-41110
JABSUU000000000	SRR12123671	DP1.3A	6,579,985	47.1	5,839,560	13.0	62	512,617	6,114	Orange plant soil, 17-4-2019	*Brevibacillus* sp.	59.10	93.65	*Brevibacillus porteri* B-41110
JABMIT000000000	SRR12105327	HB1.3	6,066,817	47.3	4,162,250	17.0	44	476,614	5,750	Orange plant soil, 17-4-2019	*Brevibacillus* sp.	53.80	93.00	*Brevibacillus formosus* DSM 9885
JABSUY000000000	SRR12141636	M2.1A	6,216,907	47.3	4,162,250	10.0	53	477,247	5,898	Maize field soil, 6-12-2018	*Brevibacillus* sp.	56.50	93.95	*Brevibacillus formosus* DSM 9885
JABSUZ000000000	SRR12141690	MS2.2	6,273,578	47.3	1,594,872	10.0	72	144,852	5,898	Maize field soil, 8-5-2019	*Brevibacillus* sp.	55.50	93.30	Brevibacillus brevis DSM 30
VEXA00000000	SRR12104985	CD3.6	4,369,550	36.8	6,766,126	25.0	13	533,701	4,321	*Brassica juncea* rhizosphere, 1-11-2018	*Lysinibacillus* sp.	31.10	85.47	Lysinibacillus varians GY32

a The cutoff value for species delimitation of ANIb (6) is defined as 96%.

b The cutoff value for species delimitation of dDDH (7) is defined as 70%.

For biomass production, colonies of a fresh culture grown on Luria-Bertani (LB) agar plates were selected. Genomic DNA was extracted using the DNeasy blood and tissue kit (Qiagen, Hilden, Germany) after growth on LB agar plates for 24 h at 37°C. The sequencing was conducted at LGC Genomics (Berlin, Germany) with an Illumina HiSeq system using paired-end 150-bp reads. Default parameters were used for all software unless otherwise specified. Reads were trimmed and filtered using fastp v0.20.1 (https://github.com/OpenGene/fastp) with default settings. *De novo* assemblies were generated by using the short-read assembler SPAdes v3.13.0 (3) (http://cab.spbu.ru/software/spades) without read correction and with normal bridging. The quality of assemblies was assessed by determining the proportion of falsely trimmed proteins by using Ideel (https://github.com/phiweger/ideel). The complete pipeline results were saved as a Snakemake file (4) and uploaded on GitHub (https://github.com/CptChiler/snakeGenome). Genome coverage of the contigs obtained was 50× on average (Table 1). Contigs were submitted to GenBank for gene annotation, which was implemented using the NCBI Prokaryotic Genome Annotation Pipeline (PGAP) v4.11 (5). The Genome-to-Genome Distance Calculator (GGDC) v2.1 provided by DSMZ (http://ggdc.dsmz.de) was used for genome-based species delineation via estimated digital DNA-DNA hybridization (dDDH) values against a reference genome. Formula 2, which is especially appropriate for analyzing draft genomes, was used (6). In addition, JSpeciesWS (http://jspecies.ribohost.com/jspeciesws) was used to determine average nucleotide identity based on BLAST+ (ANIb) values by pairwise genome comparisons (7). Accession numbers and characteristics of the genomes, including their ANIb values, are summarized in Table 1.

According to their draft genome sequences, we have assigned 49 of the isolates with potential to control plant pathogens as representatives of Bacillus altitudinis (strain BT2.2), Bacillus cereus (strains A8, A22, A24, A31, A42, HB3.1, HD1.4B, HD2.4, M2.1B, SN4.3, and TK1), Bacillus pacificus (strain SN4-1), Bacillus subtilis subsp. *subtilis* (strain GR2.1), Bacillus tequilensis (strain DL2.1), Bacillus tropicus (strains CD3.2 and SN1), Bacillus velezensis (strains A25, A35, BT2.1, BT2.4, CP5.2, CP6, CP7.1A, CP7.1C, CP7.2A, DP1.3B, DP2.2B, EG5.1A, HD1.1, HD2.2, HD2.5, HD3.1B, HD5.1, HD5.2A, KT1, MR2.1A, OL1.1, OR2.1, S1, S2, TK2, TL7, and BP1.2A), Brevibacillus parabrevis (strains HD1.4A and HD3.3A), and *Brevibacillus porteri* (strains HB1.1, HB1.2, and HB1.4B).

Ten strains, i.e., *Bacillus* sp. strains HD1.3, CD3.1A, and CD3.5, *Brevibacillus* sp. strains HB2.2, RS1.1, DP1.3A, HB1.3, MS2.1A, and MS2.2, and *Lysinibacillus* sp. strain CD3.6, could not be identified to the species level since their estimated taxonomic values and values were below the species cutoff values (GGDC, <70%; ANIb, <96%). Further research in order to characterize these novel biocontrol strains and their secondary metabolites is in progress.

### Data availability.

These whole-genome shotgun projects have been deposited in GenBank under the accession numbers listed in Table 1.
